# Potentially Extreme Population Displacement and Concentration in the Tropics Under Non-Extreme Warming

**DOI:** 10.1038/srep25697

**Published:** 2016-06-09

**Authors:** Solomon M. Hsiang, Adam H. Sobel

**Affiliations:** 1 Goldman School of Public Policy, University of California, Berkeley, USA; 2 National Bureau of Economic Research, Cambridge, Massachusetts, USA; 3 Department of Earth and Environmental Science, Columbia University, USA; 4 Department of Applied Physics and Applied Mathematics, Columbia University, USA; 5 Lamont-Doherty Earth Observatory, Columbia University, USA.

## Abstract

Evidence increasingly suggests that as climate warms, some plant, animal, and human populations may move to preserve their environmental temperature. The distances they must travel to do this depends on how much cooler nearby surfaces temperatures are. Because large-scale atmospheric dynamics constrain surface temperatures to be nearly uniform near the equator, these displacements can grow to extreme distances in the tropics, even under relatively mild warming scenarios. Here we show that in order to preserve their annual mean temperatures, tropical populations would have to travel distances greater than 1000 km over less than a century if global mean temperature rises by 2 °C over the same period. The disproportionately rapid evacuation of the tropics under such a scenario would cause migrants to concentrate in tropical margins and the subtropics, where population densities would increase 300% or more. These results may have critical consequences for ecosystem and human wellbeing in tropical contexts where alternatives to geographic displacement are limited.

It is now widely understood that ecosystems and, to some extent, human populations respond to changing climates by moving^1^,^2^,^3^,^4^,^5^,^6^,^7^,^8^,^9^,^10^,^11^,^12^,^13^,^14^,^15^,^16^,^17^,^18^,^19^,^20^,^21^,^22^,^23^,^24^,^25^. For example, butterflies^3^, marine fish^4^, and plants^5^ have been shown to move to cooler locations due to recent warming, and human populations have moved rapidly in response to the American Dustbowl^21^, as well as recent persistent warming events in Mexico^10^, Indonesia^17^, and Pakistan^16^. Prior work has identified many local factors that influence how populations may move in response to changes in their local climate^26^,^27^, such as the role of local topography or the movements of nearby competitor populations. We build on this understanding, and emphasize a constraint from planetary-scale atmospheric dynamics which may also play an important role in determining how ecosystems and human populations might move in response to climate change. We intentionally develop a simple model to highlight a single climate-biology linkage that emerges as a consequence of the earth’s sphericity and rotation.

We adopt the most basic possible starting point, a model in which populations adapt to climate change by moving such that their environmental temperature remains the same. This simple model intentionally does not capture many of the dynamics previously studied, but it is at least conceptually consistent with many empirical observations^2^,^7^,^8^,^10^,^11^,^12^,^13^,^14^,^16^,^18^,^19^. We point out here that the magnitudes of such temperature-preserving displacements, and the concentrations of populations that would necessarily result, have the potential to be extraordinary in the tropics even for magnitudes of warming that are at the low end of current projections. Because horizontal temperature gradients are small in the tropics, large displacements are generally required to achieve even small reductions in temperature. The smallness of tropical temperature gradients results from fundamental fluid mechanical constraints on the atmospheric circulation which are well understood^28^,^29^,^30^.

To clarify ideas, we imagine a topography-free planet with a zonally symmetric climate. On this planet, there are only north-south gradients in the climatological distribution of temperature *T*(*y*), where *y* is the signed distance from the equator (e.g. latitude). If we impose a small change in climatology Δ*T*(*y*), the characteristic length-scale *L* of a temperature-preserving displacement is


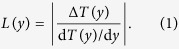


For a fixed temperature change, *L* depends inversely on the local temperature gradient d*T*(*y*)/d*y* (see Methods).

Horizontal temperature gradients in the atmosphere are closely related to horizontal pressure gradients by hydrostatic balance. Outside the tropics, relatively large horizontal pressure gradients, and thus also large temperature gradients, can be sustained because the Coriolis force can balance the pressure gradient force. Near the equator, the horizontal component of the Coriolis force becomes weak due to the small projection of the earth’s rotation vector on the local vertical. As a consequence tropical pressure and temperature gradients are much smaller than extratropical ones. Perusal of global maps of temperature on a surface of constant altitude show much larger variations at high latitudes than low^31^, no matter what season, year, or time-averaging period is chosen. Because of this, the displacement distance *L* is necessarily larger in the tropics than in the extratropics, sometimes dramatically so.

## Results

We estimate *L* in Equation 1 for a scenario in which global mean temperatures rise 2.0 °C. We compute the multi-model mean zonally-averaged climatology for twenty general circulation models collected by the Intergovernmental Panel on Climate Change (IPCC)^32^. We compare a baseline climatology *T*(*y*) computed for the immediate future 2011–2030 against a more distant climatology over 2080–2099, the period on which IPCC projections are primarily focused. In the following analysis, we separately examine surface temperatures over the oceans and over the continents since we assume that populations can only inhabit one or the other of these environments. We also limit our analysis to 50°S-50°N latitude. The polar regions require special consideration because no displacement can go further poleward than the poles themselves; furthermore, the populations of human and most species become small near the poles under the present climate.

**Figure 1:**
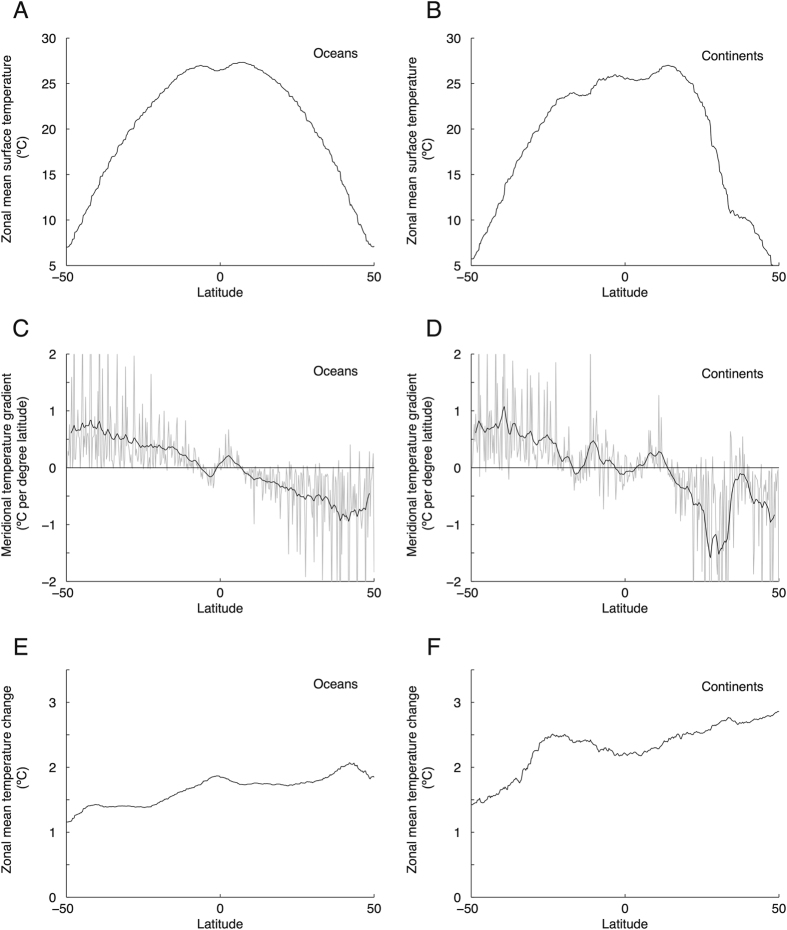
Zonally-averaged surface temperature patterns averaged over 20 climate models. (**A**) Zonal mean surface temperature *T*(*y*) of ocean pixels, 2011–2030. (**B**) Same, but for continental pixels. (**C**) Derivative d*T*(*y*)/d*y* for panel A (grey, smoothed is black). (**D**) Same, but for panel B. (**E**) Zonal mean ocean surface temperature change Δ*T*(*y*) between 2011–2030 and 2080–2099. (**F**) Same, but for continents.

In Fig. 1, panels A and B plot the initial temperature profiles *T*(*y*) for the oceans and continents, respectively, while panels C and D plot the local derivative d*T*(*y*)/d*y*, which is smoothed for clarity. As we expect, d*T*(*y*)/d*y* is basically zero close to the equator and increases in magnitude in middle latitudes. Panels E and F plot the change in temperature Δ*T*(*y*) in the 2.0° scenario, which is relatively constant over latitude except for far southern regions that warm somewhat less.

**Figure 2:**
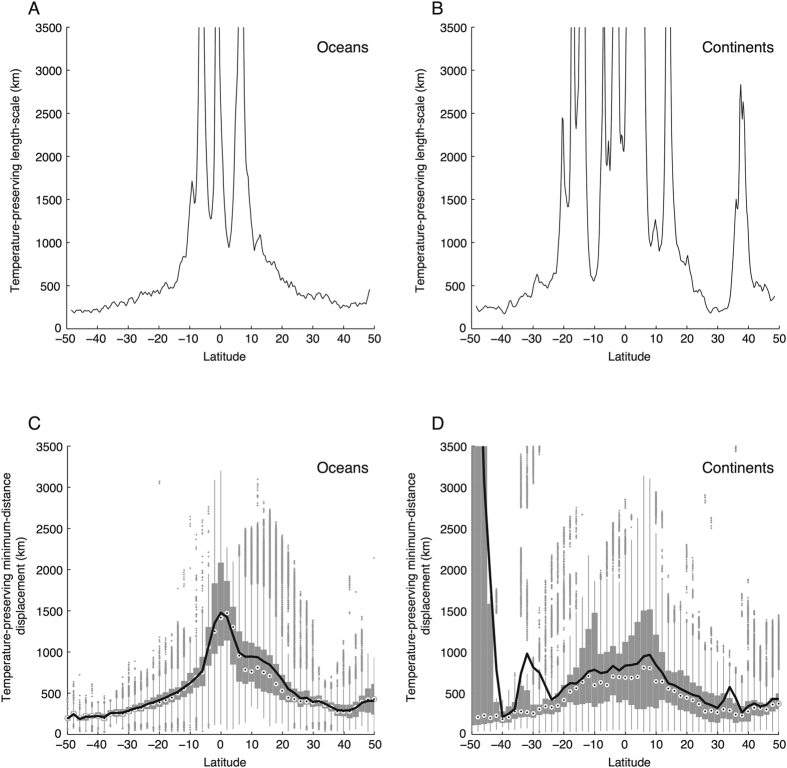
Theoretical migration length scales and actual temperature-preserving displacements are extreme in the tropics. (**A**) Length scale for temperature preserving displacement (Equation 1) in the ocean, computed with zonal mean profiles from Fig. 1. (**B**) Same, but for the continents. (**C**) Distributions of the shortest actual temperature-preserving migration for pixels in each 2° latitude bin of the oceans. Circles are medians, boxes are inter-quartile ranges, vertical lines are ranges, dots are outlying observations, and the black line connects mean values. (**D**) Same, but for continental pixels.

In panels A and B of Fig. 2, we compute the characteristic length scale *L*(*y*) using the meridional profiles shown in Fig. 1. Because the temperature change Δ*T* is roughly constant with latitude while temperature gradients approach zero near the equator, *L* increases to large values both in the tropical ocean and on tropical continents.

The zonally symmetric model in Equation 1 implicitly constrains population movements to occur along the north-south axis and assumes that local temperature gradients do not vary over the course of a migration. We relax these two assumptions by computing the shortest actual distance that populations must move to preserve their average temperature. For each pixel *i*, we locate the nearest pixel *j* that exhibits a future mean temperature that is equal to or lower than the initial temperature at *i*, subject to the constraint that *j* is not oceanic if *i* is continental and *vice versa*. We plot the distribution of these distances for each 2° latitude band in panels C and D of Fig. 2. The displacement distances thus computed tend to be substantially larger for initial positions in the tropics than for those in the middle latitudes, consistent with our simpler length scale analysis. For several latitude bands near the equator, more than 75% of oceanic locations require that populations must migrate more than 1000 km to preserve their average surface temperatures. On the continents, more than 25% of locations in a broader latitude band near the tropics require that populations move more than 1000 km. Both in the ocean and on continents, displacements exceeding 2000 km appear in a narrow band near the equator.

**Figure 3:**
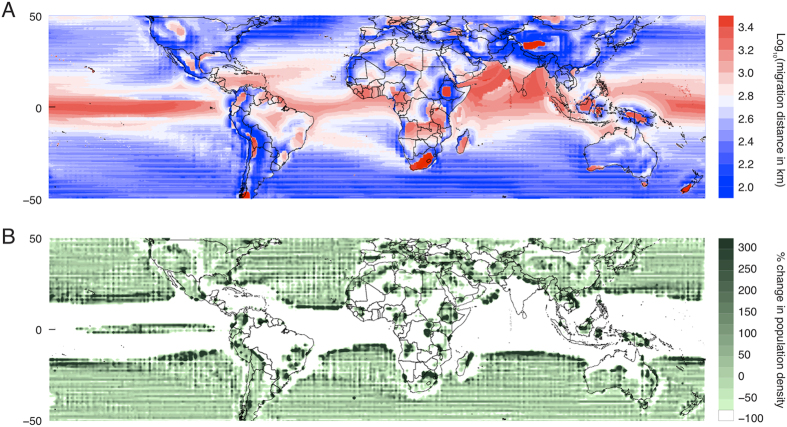
The length of minimum-distance temperature-preserving displacements and their impact on population density. The shortest temperature-preserving migration is computed for each pixel under 2 °C of global mean warming. Populations that are initially in the ocean (on land) are constrained to remain in the ocean (on land). Striped appearance over some regions occurs because the combined climate models vary in spatial resolution. (**A**) Logarithm of the minimum distance that an organism must travel to maintain the average temperature of its environment, plotted as a function of the organism’s initial location. (**B**) The percent change in population density that occurs if a hypothetical population were initially distributed uniformly over the globe and all members of that population undertake the minimum-distance temperature-preserving displacement in (**A**). Maps created by authors using Matlab.

While the structures shown in panels C and D of Fig. 2 match those in panels A and B quite well, there are some deviations that can be understood by examining the map of our calculated displacement distances shown in Fig. 3A. For example, the North-South asymmetry in the dispersion of oceanic displacements (Fig. 2C) is due to large movements required by populations initially in the north Indian Ocean, where the Asian continent prevents northward movements; and large distances arise in the southern continents (Fig. 2D) because the Southern Ocean prevents continuous southward movements from New Zealand and the southern tips of Africa, South America and Australia. The map also reveals the local influence of topography (on the continents) and coastal upwelling (in the oceans), both of which are important because these features perturb local temperature gradients relative to the zonal mean.

A logical consequence of greater displacements of tropical populations than others is the extreme concentration of populations at the margins of tropical regions. To illustrate this, we simulate a population which is initially distributed uniformly around the globe but whose members follow the temperature-preserving displacements in Fig. 3A without experiencing any population growth. The resulting population density is shown in Fig. 3B. In the middle latitudes, population densities are largely unchanged because populations at each location shift poleward at roughly the same rate, analogously to many cars all moving forward together at the same speed. In contrast, the large displacements in the tropics lead to an almost complete evacuation of the equatorial band, with the displaced populations accumulating in tropical margins where the speed of migration rapidly slows. This is analogous to the traffic jam that occurs when a highway accident brings fast moving cars to an abrupt halt. The effect on population densities in tropical margins is dramatic, in both the oceans and on the continents, as population densities climb to above 400% of their initial concentrations. If populations were actually to concentrate this quickly in what are already exceptionally arid environments, we would expect there to be many adverse consequences in both natural and human systems, such as an accelerated transmission of infectious diseases or conflict over scarce resources.

## Discussion and Conclusions

For many species (including humans), geographic displacement is only one of multiple possible adaptations to climate change, the choice of which may vary by population and context^7^,^8^,^9^,^12^. The temperature-preserving displacements presented here are likely to be employed only when less costly adaptations are unavailable^9^,^33^. In addition, some populations may not move even if they are unable to adapt along other dimensions—instead, they will simply bear the cost of elevated environmental temperatures. The tropics are already the warmest part of the planet, however, and the cost of thermal exposure may rise nonlinearly at a critical threshold^9^,^11^,^34^,^35^,^36^,^37^,^38^,^39^. As a consequence, “staying put” and enduring additional warming may be extremely harmful to many tropical species and societies. For tropical populations, temperature-preserving displacement may be simultaneously a more beneficial strategy and—because the required displacements are larger—one more difficult to execute than it is for those starting outside the tropics.

Population displacements could be in part vertical as well as horizontal, taking advantage of vertical temperature gradients in additional to horizontal ones^18^,^19^. Vertical temperature gradients are generally large in the tropics, with temperatures dropping quickly as one moves higher into the atmosphere or deeper into the ocean. Our analysis captures this effect on continents for topographic features resolved at the coarse climate model resolutions we use. In the oceans, and in continental locations where topographic features are too localized to be captured by global climate models, horizontal temperature-preserving displacements may be shorter than we calculate if populations are able to move vertically at the same time. It may not be feasible for many species to do so, however, due to other environmental constraints. Light availability decreases rapidly with depth in the oceans, and available land area declines quickly with altitude on topographic features of sufficiently fine scale to be absent from our analysis.

**Figure 4:**
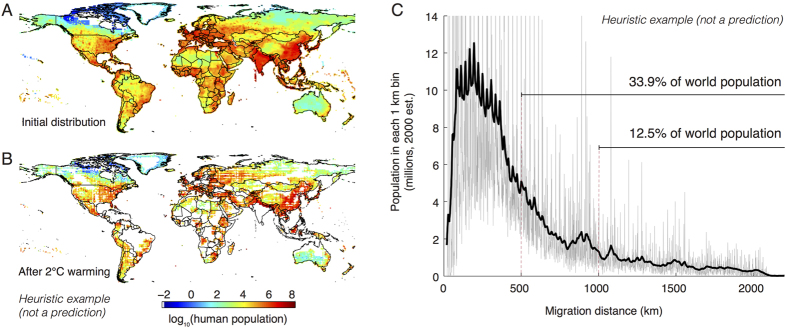
Heuristic example applying results to the global human population. To aid in visualizing the result of this analysis, temperature-preserving displacements are applied to the global distribution of the people as an illustrative thought experiment, since this is one species distribution that is familiar and well documented. Actual human migrations will certainly differ and likely will be less extreme, as people can adapt and access technologies that may allow them to avoid displacement, behaviors that are abstracted away in this analysis. (**A**) Logarithm of the current distribution of humans^41^. (**B**) The distribution of this population if all individuals undertake the displacement in Fig. 3. (**C**) Histogram with 1 km bins (grey, smoothed is black) for the minimum distance traveled by each person currently on Earth. Maps created by authors using Matlab.

Our analysis intentionally abstracts away from many of these complexities to focus on the role of weak temperature gradients in the tropics and we do not focus on any specific species since our result comes from general dynamics of the atmosphere. Nonetheless, it is instructive to develop a heuristic example by applying temperature-preserving migrations to the actual population distribution of a real species, an exercise that—while limited in numerous ways—provides some additional perspective on the structure and magnitude of the dynamics in our simplified model. For demonstration purposes, we use a specific species distribution that is both convenient and intuitive: the distribution of modern humans^41^ (Fig. 4A). Recognizing this calculation is not a prediction of actual human migrations, we relocate all currently living people according to our temperature-preserving assumption. As shown in Fig. 4B, the final population distribution is generally more concentrated, especially in the tropical margins of Latin America, Africa, and South Asia. Computing a histogram for the number of people who would migrate each distance (Fig. 4C), we find that 12.5% of the global population, most of which is currently in the tropics, would have to migrate more than 1000 km. 33.9% of the population would have to migrate more than 500 km. Imagining the tremendous cost of actually undertaking such massive spatial reorganization of the global population helps illustrate the potential importance of the dynamics we highlight here, although in the context of humans there are likely many local adaptations that are preferred to these displacements.

It is likely that some populations might migrate to conserve features of the local temperature climatology other than average temperature^8^,^9^,^12^,^19^, such as the variance of interannual temperature or maximum seasonal temperature. While displacements which preserve these statistics will differ somewhat from those we present here, the extreme behavior of the tropics is likely to persist. Tropical gradients in most of these statistics are weak for the same reason that gradients in average temperature are weak. Due to the weak Coriolis force near the equator, temperature changes throughout the tropics occur coherently across planetary spatial scales, making it difficult for surface-bound populations to move into a different temperature regime without traveling large horizontal distances.

For many species, particularly sessile species or those with limited motility, the speed of migration necessary to preserve temperature is more important than the total displacement distance^7^,^8^,^18^. For example mature forests and coral reef systems are sensitive to temperature and are much less mobile than most vertebrates, including people. In the scenario considered here, extreme displacements of 1000–2000 km must be achieved within 89 years, requiring that temperature-preserving movements have an average speed of 11.2–22.5 km yr^−1^ (1.3–2.6 m hr^−1^). These speeds substantially exceed the rate of previously observed climate-induced range shifts^1^,^4^,^5^,^6^,^19^ and prior estimates for movement under future warming that do not account for large-scale temperature gradients^18^,^40^. If maintaining their present environmental temperature is a critical adaptation to anthropogenic climate change, some tropical populations may have to migrate at unprecedented speeds over extreme distances in order to cope with relatively optimistic warming projections, given current emissions trajectories.

## Methods

For a global mean temperature change 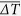, we set the average temperature changes experienced by an organism to zero


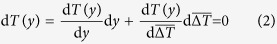


and rearrange to obtain


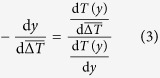


where 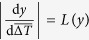, the length-scale traversed in response to the local temperature change 
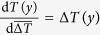
. This differential holds for relatively small displacements where the second order effect of global climate change on local temperature gradients can be assumed zero, i.e. 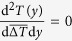. In the case of climate changes that we will consider here, the change in local temperature gradients is very small, amounting to roughly 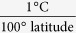 (see Fig. 1E,F).

## Additional Information

**How to cite this article**: Hsiang, S. M. and Sobel, A. H. Potentially Extreme Population Displacement and Concentration in the Tropics Under Non-Extreme Warming. *Sci. Rep.* **6**, 25697; doi: 10.1038/srep25697 (2016).
